# Robust HPV‐16 Detection Workflow for Formalin‐Fixed Cancer Tissue and Its Application for Oral Squamous Cell Carcinoma

**DOI:** 10.1002/cam4.70544

**Published:** 2025-02-20

**Authors:** Shizuka Morodomi, Akiyuki Hirosue, Akhinur Rahman, Kyotaro Nohata, Misaki Matsuo, Omnia Reda, Samiul Alam Rajib, Haruki Saito, Hiroki Takeda, Ryoji Yoshida, Masafumi Nakamoto, Masatoshi Hirayama, Kenta Kawahara, Mitsuyoshi Takatori, Yorihisa Orita, Hideki Nakayama, Yorifumi Satou

**Affiliations:** ^1^ Department of Oral and Maxillofacial Surgery, Faculty of Life Sciences Kumamoto University Kumamoto Japan; ^2^ Division of Genomics and Transcriptomics, Joint Research Center for Human Retrovirus Infection Kumamoto University Kumamoto Japan; ^3^ Department of Infectious Disease Medicine Tokyo Medical University Hospital Tokyo Japan; ^4^ University Côte d'Azur, INSERM, CNRS, Institute for Research on Cancer and Aging of Nice (IRCAN) Nice France; ^5^ Department of Otolaryngology‐Head and Neck Surgery Kumamoto University Graduate School of Medicine Kumamoto Japan

**Keywords:** genetic diagnosis, genomic analysis, head and neck cancer, human papillomavirus (HPV), multiplex PCR

## Abstract

**Background:**

Virus‐related cancers are malignancies caused by specific viruses, such as human papillomavirus (HPV), hepatitis B virus, and human T‐cell leukemia virus, contributing significantly to the global cancer burden through persistent infection and oncogenic transformation. The current study aimed to develop a robust HPV‐16 detection method for formalin‐fixed cancer specimens.

**Materials and Methods:**

To prevent false negatives resulting from DNA fragmentation, a DNA quality check step was added. Additionally, this study used multiplex polymerase chain reaction (PCR) covering the entire HPV‐16 genome to mitigate effects caused by viral sequence variation. To prove this concept, we analyzed genomic DNA extracted from oropharyngeal cancer tissues known as HPV‐16‐positive. Subsequently, the protocol was tested on oral squamous cell carcinoma (OSCC) samples in our cohort. Given the wide variation in HPV‐16 positivity in previous studies, it remains elusive how frequently HPV‐16 is positive in OSCC.

**Results:**

The results showed faint bands or smears in the multiplex PCR of 7 out of 112 cases. Droplet digital PCR confirmed variable positivity levels of HPV‐16, suggesting two scenarios of HPV‐16 positivity in cancer tissue: cancer cells derived from infected cells or only a portion being HPV‐16‐positive. Finally, we comprehensively analyzed the case and identified the integration of a deleted HPV‐16 genome into the intronic region of the host gene *TMEM94* on chromosome 17. To the best of our knowledge, this is the first evidence showing the integration of HPV‐16 in OSCC cells and providing its complete viral sequence.

**Conclusions:**

The established protocol should be applicable to various cancer tissues for analyzing the association with HPV‐16 infection.

## Graphical abstract

The relationship between HPV‐16 and OSCC may not be as prominent as in oropharyngeal cancer, at least within our cohort. The method developed in this study is not only applicable to oral cancer. This method's utility and versatility position it as a valuable resource for elucidating virus–cancer associations in the future.

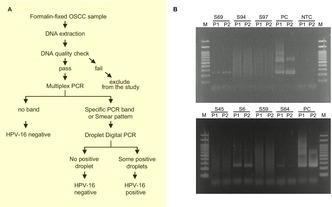

## 1 Introduction

Oral cancer accounts for approximately 50% of head and neck cancers, with over 90% of oral cancers being oral squamous cell carcinoma (OSCC). Common sites of occurrence include the tongue, upper and lower gingiva, buccal mucosa, floor of the mouth, hard palate, and lips. Despite advancements in diagnosis and treatment in recent years, the survival rate of OSCC patients has not significantly improved over the past 30 years. Of particular concern is the poor prognosis for advanced OSCC patients, with a 5‐year survival rate of approximately 50% [1]. Smoking, alcohol consumption, genetic factors, chronic irritation, and viruses are among the primary causes of oral cancer [2, 3]. These multifactorial elements interact synergistically to contribute to carcinogenesis [4].

Among various oncogenic viruses, human papillomavirus (HPV) is most frequently associated with head and neck cancers. Previous reports indicate that 50% and 70% of oropharyngeal cancers in Japan and the United States are HPV‐related, respectively [5]. There are over 120 types of HPV, with approximately 15 high‐risk types, including HPV‐16 and HPV‐18, associated with cancer, of which HPV‐16 is particularly linked to oropharyngeal cancer [6]. Structurally, HPV‐16 is a double‐stranded circular DNA virus comprising about 8000 base pairs, with early genes E1, E2, E5, E6, and E7 and late genes L1 and L2. The E6 and E7 genes are said to be crucial for carcinogenesis [7, 8]. In recent years, radiation therapy has shown promising results in HPV‐related oropharyngeal cancer, leading to favorable outcomes [9]. Various methods exist for confirming HPV infection, with p16 immunostaining being commonly used as a surrogate marker in clinical settings.

There are various methods for detecting HPV‐16, including conventional polymerase chain reaction (PCR), quantitative PCR (qPCR), droplet digital PCR (ddPCR), and in situ hybridization of DNA or E6/E7 mRNA [10]. In previous reports, when performing conventional PCR using primers for the L1 region on formalin‐fixed paraffin embedded (FFPE) or frozen tissue, the reported HPV‐16 positivity rates ranged from 4% to 32% [11, 12, 13, 14] and from 2% to 61% [15], respectively. The variability in HPV‐16 positivity rates can be attributed to the proximity of the oropharyngeal and oral regions, which leads to variations in defining the boundary, as well as the lack of a universal HPV detection method. While p16 immunohistochemistry is a useful method for detecting HPV16 in oropharyngeal cancer, it has been reported to be ineffective in oral cancer [16]. Additionally, compared to HPV‐16‐positive oropharyngeal cancer, much remains unclear about the involvement of HPV‐16 in carcinogenesis and treatment sensitivity [11, 12, 13, 14].

Therefore, this study aimed to establish a reliable HPV‐16 detection method for oral cancer by considering the characteristics of the HPV‐16 sequence variability and using multiplex PCR to cover the entire HPV‐16 genome. Moreover, our work investigated the HPV‐16 positivity rate in oral cancer.

## 2 Material and Methods

### 2.1 Study Design and Specimen Collection

This study included a cohort of 127 patients diagnosed with OSCC who visited Kumamoto University Hospital between 2015 and 2022 at the Department of Oral and Maxillofacial Surgery, for whom OSCC biopsy and surgical samples were available. The formalin‐fixed paraffin‐embedded (FFPE) tissue samples were handled following the national guidelines for genomic medical care in Japan, ensuring they met the specified criteria. After DNA extraction, a quality check was performed, and 112 samples passed the criteria and were used for further analysis. Tissue samples of oropharyngeal squamous cell carcinoma (OPSCC) were included in the study as positive control samples, and they were also resected at the Department of Otolaryngology‐Head and Neck Surgery of Kumamoto University Hospital (Kumamoto, Japan) and used in the present study. FFPE samples of tumor were from three to four slices with a 4 μm thickness. DNA was extracted from samples according to a protocol using the Gene Read DNA FFPE Kit (Qiagen, Hilden, Germany) (Cat #56404). The extracted DNA concentration was measured using NanoDrop. The present study followed the guidelines of the Ethics Committee of Kumamoto University (Ethics Committee approval number 1427, No. 418).

### 2.2 Qualitative Analysis (Genotyping PCR)

Conventional genotyping PCR was performed using p16‐positive oropharyngeal cancer. Details can be found in the Supporting Information.

### 2.3 DNA Quality Check

The quality of the DNA for all samples was assessed, as reported previously [17]. Details can be found in the Supporting Information.

### 2.4 Establishment of a Multiplex PCR Method Covering the Entire HPV16 Length

Multiplex PCR was designed using the Multiplex primer design site Primal Scheme [18]. A 47‐primer set was designed based on the HPV‐16 sequences of Japanese patients with HPV‐16‐positive cancer (101 cases shown in Table S1). Primer sequences are shown in Table S2. The 47 sets of primers were divided into odd (pool 1) and even (pool 2) groups. PCR reaction conditions were 95°C for 2 min using Phusion Plus PCR Master Mix (Thermo Fisher), 5 cycles of 98°C 5 s, 62°C 10 s, 68°C 20 s, 5 cycles of 98°C 5 s, 60°C 10 s, 68°C 20 s, 5 cycles of 98°C 5 s, 58°C 10 s, 68°C 20 s, 5 cycles of 98°C 5 s, 56°C 10 s, 68°C 20 s, 20 cycles of 98°C 5 s, 54°C 10 s, 68°C 20 s, followed by 68°C 5 min.

### 2.5 DNA Sequencing by Next‐Generation Sequencing (NGS)

Multiplex PCR amplicons (pool1 and pool2) were used for the NGS library preparation. Details can be found in Supporting Information.

### 2.6 Absolute Quantification of Viral DNA by ddPCR

To determine the HPV‐16 copy number, ddPCR was performed for six patient tissues, of which a single band or smear were obtained by multiplex PCR; S69 was excluded from the multiple region ddPCR due to insufficient samples. Six HPV‐16 positive OPSCC specimens were tested, along with UM‐SCC‐47 as a positive control and a non‐template control (NTC). Absolute quantification of the host cell genome copy number and HPV copy number was used to calculate the percentage of infected cells in the tissue samples. ddPCR was performed using master mix and oil for EvaGreen (BIO‐RAD) (cat #1864034) and Digital PCR machine (QX200 Droplet Digital PCR System, Bio‐rad, USA). PCR conditions were used as per manufacturers' protocol: 95°C for 10 min, followed by 39 cycles of 94°C for 30 s, 58°C for 60 s, then 98°C for 10 min. Multiplex primers' set 1/25/36/45 in the E2/L2/L1/E6 region was used. Analysis of the ddPCR data was performed using the ddPCRquant R package. In brief, baseline correction for NTC is performed, followed by threshold line calculation. Sample processing that applies the calculated threshold is finally performed [19].

### 2.7 Viral DNA‐Capture‐Seq

Viral DNA‐capture‐seq with biotinylated HPV‐16 probes (Table S3) was performed for the cell line, UM‐SCC‐47, and FFPE sample S6 in a similar way as previously reported [20, 21, 22]. Details can be found in Supporting Information.

## 3 Results

### 3.1 Factors That Could Potentially Impede the Accuracy of HPV‐16 Detection in Formalin‐Fixed Tissue Samples

**FIGURE 1:**
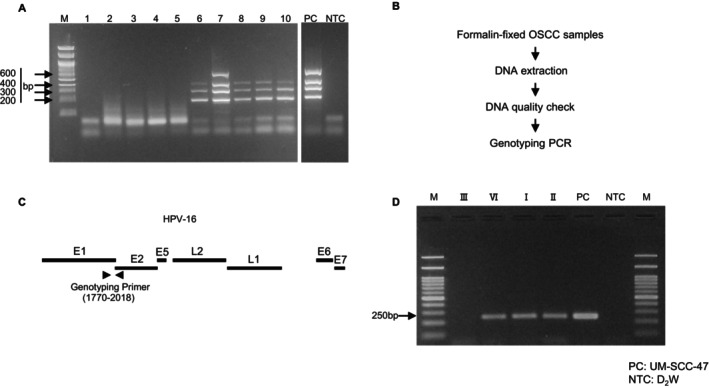
Conventional PCR method capacity to identify HPV‐16 positivity in formalin‐fixed samples. (A) Agarose gel pictures depicting qualitative PCR results of RAG1 exon2 (200 bp), PLZF exon1 (300 bp), AF4 exon11 (400 bp), and AF4 exon3 (600 bp) regions for ten OSCC samples (1–10) at the time of the biopsy. HPV‐16‐infected cell line (UM‐SCC‐47) was used as a positive control (PC) and D_2_W as a non‐template control (NTC). Samples with the expected band size detected above 300 bp were considered of good DNA quality and used for the next step (112/127 samples). A 100 bp molecular marker (M) was used throughout the study. (B) Workflow for DNA quality assessment and genotyping for formalin‐fixed OSCC samples. (C) Schematic representation of genotyping PCR for HPV‐16. The genotyping primer pair was designed for the E2 region (1770–2018) with an amplicon expected size of 248 bp. (D) Agarose gel pictures showing results of OPSCC samples genotyping PCR. UM‐SCC‐47 was used as a PC and D_2_W as a NTC. The expected band size of ~250 bp was detected in samples VI, I, and II as well as PC.

Given the wide variability in reported HPV‐16 positivity in OSCC [11, 12, 13, 14], we hypothesized that certain factors might introduce bias into the analysis. Most of the previous studies utilized PCR to analyze the presence of HPV‐16 in cancer tissues using genomic DNA (gDNA) extracted from formalin‐fixed samples previously diagnosed as cancerous through pathological examination. A significant concern here is the potential of the high fragmentation of gDNA due to excessive formalin fixation, which could lead to false negatives in HPV‐16 detection by PCR. The quality of gDNA was assessed by conducting PCR targeting host genes of different sizes according to previous reports [17]. It was found that some gDNAs extracted from OSCC tissues were negative in the PCR examination (Figure 1A). To avoid this issue, only the gDNA samples that passed the DNA quality check examination were included (Figure 1B).

Another concern was the virus sequence variation, as certain viruses found in cancer cells often accumulate mutations or deletions in their genomes (e.g., EBV and HTLV) [20, 23, 24]. Virus antigens are typically identified as foreign by the immune system. Thus, cells with mutated or deleted viral genomes may evade immune detection and subsequently gain an advantage in cancer development. This variability in viral genome sequences can introduce technical biases in the evaluation of HPV‐16 positivity. Mutations or deletions within the primer binding sites of the PCR assay designed to detect HPV‐16 can lead to negative PCR results, even when HPV‐16 is present in the tissue. To prove this concept, we analyzed gDNA extracted from OPSCC tissues known as HPV‐16‐positive. After performing conventional PCR, a false‐negative case was found. Although all tissues tested positive for HPV‐16, one tissue yielded a negative result in the HPV‐16 PCR due to a mutation in the primer binding site (Figure 1C,D; Figure S1, Table S5).

### 3.2 Establishment of a Comprehensive PCR Method for HPV‐16 DNA Detection

**FIGURE 2:**
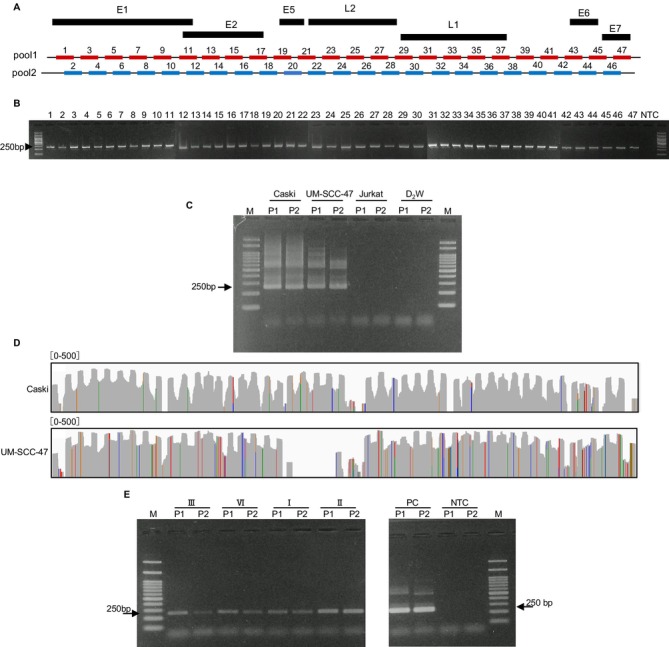
Establishment of a comprehensive PCR method for HPV‐16 DNA detection. (A) Schematic representation of multiplex PCR (Tiling PCR) primer design. The 47 primer sets were designed to cover the entire length of the HPV‐16 genome. Each primer set was designed to amplify a 250 bp region of the HPV‐16 genome. Pool 1 and Pool 2 indicate the odd and the even numbers of the 47 primer sets, respectively. (B) Two percentage agarose gel images depicting the band size of multiplex PCR primer sets. A positive control cell line (Caski) was used, and all 47 primer sets showed an expected PCR band size (250 bp). (C) Agarose gel picture band size retrieved from multiplex PCR for HPV‐16 amplification using Pool 1 (P1) and Pool 2 (P2) primer sets in Caski and UM‐SCC‐47 cell lines as positive controls. Jurkat T‐cell line and D_2_W were used as negative controls. (D) IGV coverage track of HPV‐16 aligned reads retrieved from NGS of PCR amplicons P1 and P2 shown in (C) for Caski and UM‐SCC‐47 cell lines. (E) Agarose gel electrophoresis results of multiplex PCR of gDNA extracted from OPSCC formalin‐fixed samples. P1 and P2 primer sets were used per sample. The UM‐SCC‐47 was used as a positive control (PC) and D_2_W as a non‐template control (NTC).

To prevent false negatives caused by variability in viral genome sequences, we devised a multiplex PCR protocol targeting the entire HPV‐16 genome. Drawing from previously reported HPV‐16 sequences (Table S1), a consensus sequence was generated. We then employed algorithms for multiplex PCR [18], resulting in the design of 47 PCR sets spanning the entire HPV‐16 genome (Figure 2A). The efficiency and specificity of each individual PCR were confirmed by observing single bands at the expected positions (Figure 2B). Next, we conducted multiplex PCR by dividing the 47 PCRs into two reactions, as depicted in Figure 2A, to avoid overlapping PCR amplification and prevent competition between PCR reactions. gDNA from Caski and UM‐SCC‐47 cell lines, known to contain HPV‐16, were used as positive controls, and gDNA from the Jurkat T‐cell line, which does not contain HPV‐16, was used as a negative control. Gel electrophoresis result showed a band at the expected position (250 bp) and an additional faint band at a larger size position (600 bp) both in Caski and UM‐SCC‐47 but not in the Jurkat T‐cell line (Figure 2C). Since the copy number of viral DNA is high in Caski and UM‐SCC‐47, we observed a dense pattern of the bands. To ascertain whether the multiplex PCR could amplify a broad region of HPV‐16, deep sequencing using the PCR products from HPV‐16‐positive cell lines, Caski, and UM‐SCC‐47 was performed. The sequencing data were visualized using Integrated Genomic Viewer (IGV) (Figure 2D). Caski exhibited over 90% coverage of the entire HPV‐16 genome, while UM‐SCC‐47 showed over 85% coverage. Furthermore, the newly developed multiplex PCR successfully detected HPV‐16 in all gDNA extracted from formalin‐fixed cancer tissue, including sample III, where false‐negative results were observed in the single PCR (Figures 1D and 2E and Table S5). This suggests that the HPV‐16 detection method developed in this study provides more accurate evidence of HPV‐16 positivity than conventional single PCR methods.

### 3.3 Analysis of HPV‐16 Positivity in a Formalin‐Fixed OSCC Specimen

**FIGURE 3:**
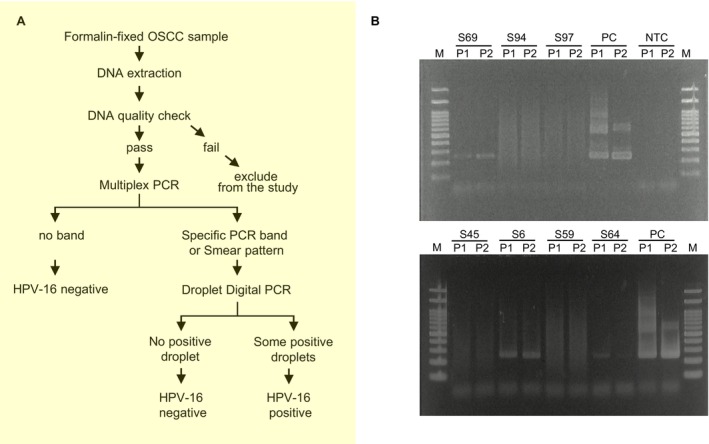
HPV‐16 detection in formalin‐fixed tissue of oral squamous cell carcinoma. (A) Analysis workflow for formalin‐fixed OSCC samples. (B) Two percentage agarose gel picture depicting band size of multiplex PCR primer pools amplifying HPV‐16 in OSCC samples. The UM‐SCC‐47 was used as a positive control (PC) and D_2_W as a non‐template control (NTC).

To obtain accurate and less biased HPV‐16 positivity in OSCC, a new detection protocol was established for clinical specimens in this study cohort. The workflow of the analysis is shown in Figure 3A. We enrolled 127 cases (Table S4) and conducted a DNA quality check using PCR, as illustrated in Figure 1B. The inclusion criteria of this study were set as: a sample was considered to have a good DNA quality if its gDNA showed a PCR band with 300 bp or more in length and therefore used in the next step. Other samples with DNA quality below that were excluded from this study.

Next, multiplex PCR was performed using 112 cases of DNA, and various electrophoretic patterns were obtained. For OSCC samples, most cases (105/112) showed neither bands nor smears, while four cases exhibited smear bands and three cases displayed faint bands (Figure 3B and Figure S2). We first defined samples with neither smear nor band as HPV‐16‐negative OSCC. Based on these results, the samples showing a single band or smear were categorized as candidates for HPV‐16‐positive. Clinical characteristics of the seven candidate samples, such as age, gender, and tumor stage, are shown in Table 1.

**TABLE 1:** Clinical information of HPV‐16‐positive candidate cases.

Number	Age	Gender	Region	cTNM classification	Smoking	Alcohol consumption
S6	74	M	Tongue	T2N0M0	ー	ー
S45	52	F	Tongue	T1N0M0	+	+
S59	80	M	Buccal mucosa	T3N3bM0	+	ー
S64	54	F	Tongue	T1N0M0	ー	+
S69	84	F	Maxillary gingiva	T1N0M0	ー	ー
S94	84	M	Tongue	T3N2bM0	ー	ー
S97	59	M	Tongue	T4aN1M0	+	+

*Note:* Clinical information includes age, gender, sampling site, cTNM (T: primary tumor, N: regional lymph nodes, M: distant metastasis), tobacco smoking, and alcohol consumption.

### 3.4 Verification of the Multiplex PCR Result by ddPCR

**FIGURE 4:**
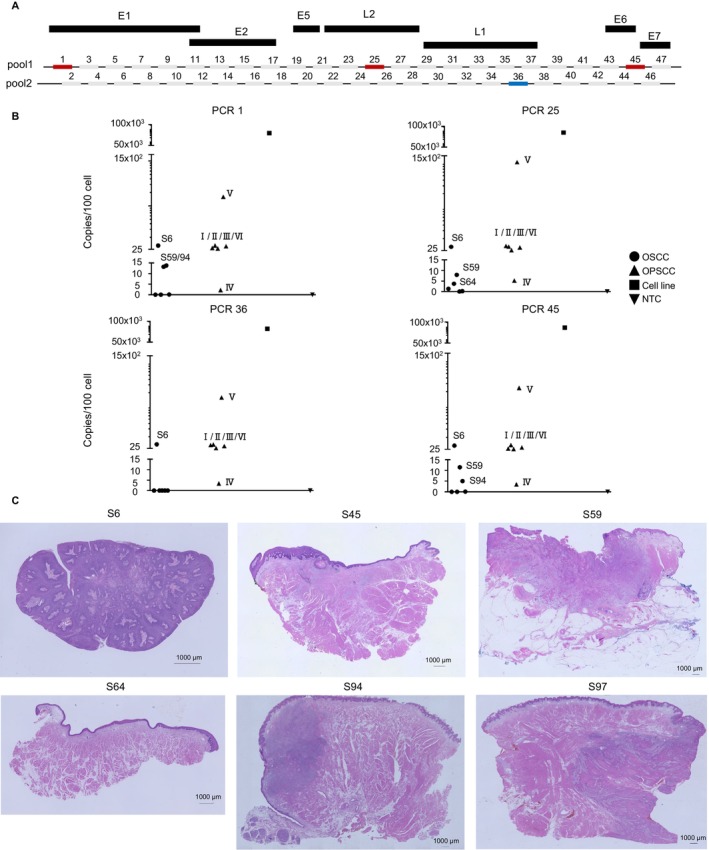
Verification of the multiplex PCR result by droplet digital PCR (ddPCR). (A) Schematic representation of multiplex PCR primer design with the primer set used for ddPCR highlighted in red (Pool 1) or blue (Pool 2). (B) Results of HPV‐16 detection by ddPCR. Each plot represents one primer set tested for six OSCC samples, six OPSCC samples, UM‐SCC‐47 cell line, or NTC. Results are presented as the number of HPV‐16 copies/100 cells, as calculated by the Albumin gene copy number as a housekeeping gene. (C) Histopathological section stained with hematoxylin and eosin for OSCC HPV‐16 candidate samples (1000 μm scale bar).

We observed smears or faint bands in the HPV‐16 multiplex PCR; however, it was difficult to distinguish between specific and non‐specific PCR amplification. To validate this result, additional ddPCR using four PCR primer sets from the HPV‐16 multiplex PCR was conducted (Figure 4A). In general, more than 10,000 individual PCRs were performed using the ddPCR method, allowing us to calculate the absolute copy number of target DNA even in a sample containing a low number of target DNA. This approach provides concrete evidence to distinguish whether the sample DNA contains HPV‐16 DNA or not. To ensure consistency, OPSCC samples were analyzed as controls of HPV‐16‐positive human cancer tissue. In the ddPCR for the albumin gene, similar positivities was observed among different samples, confirming that the amount of input DNA was equivalent across the analyzed samples (Figure S3).

The ddPCR results with four PCRs targeting different HPV‐16 genomic regions consistently showed similar positivities across all OSCC samples tested. OSCC sample S6 exhibited positive droplets clearly in all four PCR reactions. Conversely, OSCC samples, including S45, S59, S64, S94, and S97, were not all positive for four PCRs, although a few positive droplets were observed in some PCRs (Figure 4B and Figure S3). Based on the ddPCR results, the HPV‐16 copy number per 100 cells in each tissue was calculated. We found that the average copy number was 84.7, 0.3, 8.2, 0.9, 4.7, and 0.1 for samples S6, S45, S59, S64, S94, and S97, respectively (Figure 4B and Figure S3 and Table 2).

**TABLE 2:** ddPCR results for OSCC and OPSCC samples.

Patient ID/sample	Primer set				
	Albumin	PCR 1	PCR 25	PCR 36	PCR 45
S6	40.4 (20.2)	83.1	83.1	89.7	82.9
S45	92.8 (46.4)	0	1.3	0	0
S59	20.2 (10.1)	13.2	7.9	0	11.5
S64	38.6 (19.3)	0	3.7	0	0
S94	18.0 (9.0)	13.8	0	0	5.0
S97	194.6 (97.3)	0.1	0.2	0	0.1
I	56.9 (28.5)	46.2	96.7	76.1	38.4
II	72.9 (36.4)	85.8	86.5	85.9	92.0
III	50.9 (25.5)	38.1	30.8	28.6	31.0
IV	47.1 (23.5)	2.2	5.2	3.4	3.5
V	32.6 (16.3)	842.5	1402	834.5	968.6
VI	40.9 (20.4)	74.2	73.5	62.3	53.6
UM‐SCC‐47	13.6 (6.8)	79573.7	85037.5	82362.1	86666.1
D_2_W	0	0	0	0	0

*Note:* The number of infected cells was estimated by determining the viral copy number of HPV‐16 relative to the copy number of the Albumin gene, assuming that each cell contains two copies of the Albumin gene. The Albumin gene copy number was expressed per 1 µL of the PCR reaction, whereas the HPV‐16 copy number was expressed per 100 cells.

So far, OSCC samples have been analyzed with the newly developed HPV‐16 detection protocol. This protocol includes quality checks of gDNA, multiplex PCRs covering the entire HPV‐16 genome, and absolute quantification of the HPV‐16 genome by ddPCR. However, we encountered difficulties in distinguishing between positive and negative cases such as S45 and S64. Theoretically, there are at least three categories of HPV‐16‐positive cancer: (i) Cancer cells in cancer tissues fully infected with HPV‐16, (ii) cancer cells in cancer tissues partially infected with HPV‐16, and (iii) non‐cancerous cells within cancer tissue infected by HPV‐16. We termed the first scenario as “HPV‐16‐positive cancer in a narrow sense”. In contrast, the latter two scenarios were termed as “HPV‐16‐positive cancer in a broad sense”. In a narrow sense, HPV‐16‐positive cancer refers to cancer cells themselves being infected with HPV‐16, resulting in a high viral copy number per cell, as observed in OSCC S6. Conversely, in a broad sense, HPV‐16‐infected cells are present in the cancer tissue but are not in most cancer cells. In this situation, the viral copy number per cell would be low, as seen in OSCC S45, S59, S64, S94, and S97. This broader definition may also qualify as HPV‐16‐positive cancer. To ensure that cancer cells were the predominant population in the formalin‐fixed cancer tissue used for gDNA extraction, we re‐evaluated pathological findings to determine the proportion of cancer cells in the tissue. There was a wide variation in the proportion of cancer regions within each tissue, ranging from 5% to 90% (Figure 4C). Taken together, these data indicate that S6 was HPV‐16‐positive cancer in the narrow sense, whereas S45, S59, S64, S94, and S97 were HPV‐16‐positive cancer in the broader sense. Since the cancer region was limited in the samples of S45 and S64, there was a possibility that S45 and S64 might also be HPV‐16‐positive in the narrow sense. We endeavored to accurately distinguish between HPV‐16‐positive and ‐negative cases, yet there were still some instances that proved difficult to determine.

### 3.5 Characterization of the HPV‐16 Genome in the OSCC Sample by Viral DNA‐Capture‐Seq

**FIGURE 5:**
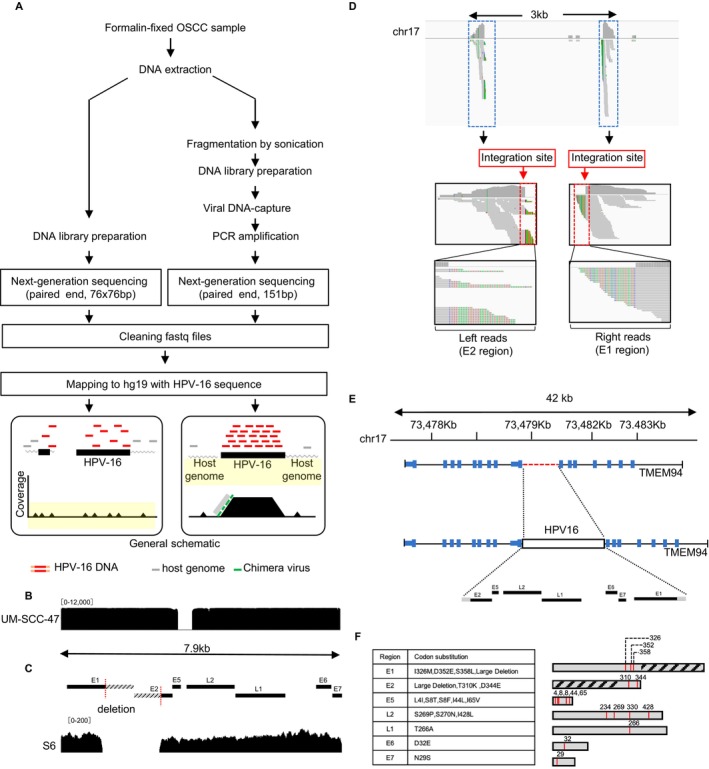
Characterization of the HPV‐16 genome in the OSCC sample by viral DNA‐capture‐seq. (A) Experimental workflow of HPV‐16 DNA‐capture‐seq for the HPV‐16 sample. An enrichment step using a DNA probe was introduced to increase the detection efficiency of HPV‐16. The general schematic figure compares the different efficiencies of HPV‐16 detection before and after treatment. (B) DNA‐capture‐seq result for UM‐SCC‐47 cells. The IGV coverage track of HPV‐16‐aligned reads is shown. (C) DNA‐capture‐seq result for the S6 sample. The upper panel shows a schematic representation of HPV‐16 genomic regions with the identified deletions in the E1 and E2 regions. The middle panel demonstrates the IGV coverage track of HPV‐16‐aligned reads. (D) IGV profile of sequencing reads obtained from S6 after alignment to hg19 and HPV‐16 sequence as an additional chromosome. Host sequences derived from virus–host chimeric reads were aligned to the shown region in chromosome 17 and denote the HPV‐16 integration site. A deletion of 2.7 kb was observed in the host genome. (E) Schematic representation of HPV‐16 integration in the intronic region of *TMEM94*. The upper panel shows the location and size of the *TMEM94* gene. The middle panel shows the depicted deletion in the *TMEM94* gene. The lower panel demonstrates the subsequent insertion of the HPV‐16 provirus. (F) Demonstrative bars representing each coding region of HPV‐16 with highlighted mutation sites (red bars), and deletions (striped bars) as deducted from the NGS data of S6. Codon substitution information is shown in the table corresponding to each region.

To characterize the entire HPV‐16 genome, this study investigated sample S6, which exhibited a high viral DNA load per 100 cells, using a technique called viral DNA capture sequencing (capture‐seq). Initially, we generated tiling DNA probes for the entire HPV‐16 genome by following a method previously described for HIV and HTLV‐1 [20, 21]. Each probe is 120 bp in length and biotinylated. Subsequently, after synthesizing the DNA library, which contains fragmented DNA from the sample, the library was mixed with the HPV‐16 probes, leading to the enrichment of HPV‐16 sequences through Streptavidin. Typically, viral DNA capture‐seq allows for the enrichment of viral genomes by 1000–10,000 times compared to the initial concentration of viral DNA before enrichment (Figure 5A), enabling us to obtain comprehensive viral sequence information.

To validate the HPV‐16 DNA capture‐seq method, the UM‐SCC‐47 cell line was analyzed and there was good coverage of HPV‐16 gDNA except for the deleted region (Figure 5B). Conducting HPV‐16 capture‐seq with the S6 DNA library successfully enriched viral DNA, with viral reads reaching 10,000 out of a total of 200,000 reads (5.0%, Figure 5C). Theoretically, the proportion of viral reads was approximately 2000 times higher than the initial proportion. Notably, we observed an approximately 1.8 kb deletion from the E1 to E2 region. Next, we analyzed virus–host chimera reads to investigate whether HPV‐16 was integrated or not (Figure 5D). The results revealed that the virus was integrated into the intronic region of the host gene *TMEM94* on chromosome 17, oriented in an opposite direction to the host genome (Figure 5E).

Moreover, the host genome exhibited a 2.4 kb deletion, suggesting that the host genome was deleted upon HPV‐16 integration into the host region. Further analysis of the HPV‐16 coding sequence within the integrated viral genome revealed that the E1 and E2 proteins were truncated due to a large deletion. However, the L1, L2, E5, E6, and E7 proteins were relatively conserved, exhibiting only a few amino acid changes compared to the reference sequence (Figure 5F). Given that the coding sequence and the transcriptionally regulatory regions of the oncogenic HPV‐16 proteins E6 and E7 were conserved, the integrated HPV‐16 viral genome theoretically has the potential to produce these proteins, thereby potentially contributing to the oncogenic process of oral cancer development. These findings collectively demonstrate that HPV‐16 was integrated into a certain case of the OSCC genome, providing evidence to elucidate the relationship between HPV‐16 and OSCC development.

## 4 Discussion

There is considerable variation in HPV‐16 positivity among previous reports on OSCC [11, 12, 13, 14]. This study aimed to develop a comprehensive and accurate method for analyzing HPV‐16 positivity using gDNA extracted from formalin‐fixed cancer tissue. To establish a reliable protocol, oropharyngeal cancer tissues with known HPV‐16 positivity were used as reference samples. By implementing DNA quality checks and multiplex PCR targeting multiple regions of the HPV‐16 genome, this study successfully developed a method that can more accurately evaluate the presence of HPV‐16 in cancer tissue compared to conventional protocols. To validate the efficiency of the method, we applied it to clinical formalin‐fixed tissue samples diagnosed as OSCC.

During our investigation of OSCC, we realized that there are HPV‐16‐positive cancer cells either in a narrow sense or in a broad sense. This different definition of HPV‐16 positiveness in cancer tissue may explain a wide variation of HPV‐16 positivity in OSCC in previous studies [11, 12, 13, 14]. To distinguish between these situations, we conducted absolute quantification of HPV‐16 DNA using ddPCR on available OSCC tissues positive in HPV‐16 multiplex PCR. The results showed that one out of six available samples contained a high amount of HPV‐16 DNA (more than 10 copies per 100 cells), indicating the presence of cancer cells infected with HPV‐16. There are three states of HPV genome in head and neck cancer reported previously, including episomal‐only state, an integrated state, and episomal virus–host chimeric DNA state [25, 26]. Based on the pattern of virus–host chimeric reads mapped to human chromosomes (Figure 5D,E), the case we found in this study has an integrated state of the HPV‐16 genome. To distinguish these different situations, viral DNA‐capture‐seq was performed, revealing the integration of HPV‐16 into the OSCC cell genome same as previously reported in difference HPV‐16‐positive cancer [27]. This study provides concrete evidence of HPV‐16 integration and its entire viral sequence in OSCC cells, a first in the field.

The OSCC case with HPV‐16 integration was identified as a mass region at the margin of the tongue. Biopsy and histological analysis confirmed it as tongue cancer. The current study explored the potential effect of the integrated HPV‐16 genome on cancer development. The oncogenesis of HPV‐16, particularly in cervical and oropharyngeal cancers, is well understood, with viral oncoproteins E6 and E7 playing key roles [7, 8]. The current study analysis revealed deletions in the HPV‐16 genome, indicating the production of truncated forms of E1 and E2 proteins. However, E6 and E7 coding regions remained conserved, suggesting their potential expression in cancer cells and contribution to oncogenic processes. In addition, we analyzed six HPV‐16‐positive samples with ddPCR and found five of them contained low amounts of HPV‐16 DNA (approximately 1 copy per 100 cells or less), indicating that only a part of cancer cells or surrounding non‐cancer cells are infected with HPV‐16. This study suggests that the relationship between HPV‐16 and OSCC may not be as significant as in oropharyngeal cancer, at least in our cohort. However, given the increasing risk of HPV‐16 infection in oral mucosal cells due to recent human behavior, continued attention to this topic is warranted [28]. Further investigation is needed to understand the relationship between HPV‐16 infection and OSCC development.

Various viruses, including EBV, HTLV, HBV, and HPV, are implicated in human cancer, contributing to approximately 30%–40% of all cancer cases [20, 23, 24]. Advances in NGS have led to decreased costs, making techniques such as whole‐exome sequencing [29, 30], whole‐genome sequencing [31], and nucleic acid testing more accessible for cancer diagnosis. This progress enables comprehensive exploration of genetic mutations and the involvement of pathogenic microorganisms, such as viruses, in cancer development. However, cancer exhibits significant diversity on a case‐by‐case basis, particularly in cancers with late onset, like oral cancer, where the cancer genome displays substantial heterogeneity. Detailed investigation into the involvement of pathogenic microorganisms, whether through direct or indirect mechanisms, remains essential for each individual case. The highly accurate and comprehensive HPV‐16 virus detection method established in our study using formalin‐fixed specimens for oral cancer can be applied to other cancers suspected of HPV‐16 involvement, including esophageal, gastric, skin, colorectal, and lung cancers.

In summary, the present study results indicate that the relationship between HPV‐16 and OSCC may not be as prominent as in oropharyngeal cancer, at least within our cohort. However, given the rising risk of HPV‐16 infection in oral mucosal cells, ongoing monitoring of this issue is crucial. Furthermore, the method developed in this study is not only applicable to oral cancer but also to various other cancer types, making it a valuable tool for investigating the association between viruses and cancer. This method's utility and versatility position it as a valuable resource for elucidating virus–cancer associations in the future.

## 5 Limitations of This Study

The DNA available in most samples, especially biopsy samples, was quite limited. Thus, we did not conduct a statistical analysis comparing HPV‐16 detection efficiency between the conventional PCR method and the new method established in this study. However, considering the comprehensiveness of the new method, it is theoretically better than the conventional approach. We demonstrated the concept by analyzing oropharyngeal cancer as an example. Further studies are needed to analyze other cancers highly associated with HPV, such as cervical cancer, to statistically validate the advantage of the new method compared to the conventional one.

## Author Contributions

**Shizuka Morodomi:** data curation (equal), formal analysis (equal), investigation (equal), methodology (equal), visualization (equal), writing – original draft (equal), writing – review and editing (equal). **Akiyuki Hirosue:** conceptualization (equal), data curation (equal), funding acquisition (equal), project administration (equal), resources (equal), supervision (equal), validation (equal), writing – review and editing (equal). **Akhinur Rahman:** data curation (equal), formal analysis (equal), investigation (equal), validation (equal), writing – review and editing (equal). **Kyotaro Nohata:** formal analysis (equal), investigation (equal), methodology (equal), writing – review and editing (equal). **Misaki Matsuo:** data curation (equal), formal analysis (equal), investigation (equal), supervision (equal), writing – review and editing (equal). **Omnia Reda:** data curation (equal), formal analysis (equal), supervision (equal), validation (equal), visualization (equal), writing – original draft (equal), writing – review and editing (equal). **Samiul Alam Rajib:** investigation (equal), methodology (equal), writing – review and editing (equal). **Haruki Saito:** investigation (equal), resources (equal), writing – review and editing (equal). **Hiroki Takeda:** resources (equal), supervision (equal), writing – review and editing (equal). **Ryoji Yoshida:** resources (equal), supervision (equal), writing – review and editing (equal). **Masafumi Nakamoto:** resources (equal), supervision (equal), writing – review and editing (equal). **Masatoshi Hirayama:** resources (equal), supervision (equal), writing – review and editing (equal). **Kenta Kawahara:** resources (equal), supervision (equal), writing – review and editing (equal). **Mitsuyoshi Takatori:** data curation (equal), formal analysis (equal), writing – review and editing (equal). **Yorihisa Orita:** project administration (equal), resources (equal), supervision (equal), writing – review and editing (equal). **Hideki Nakayama:** funding acquisition (equal), project administration (equal), resources (equal), supervision (equal), validation (equal), writing – review and editing (equal). **Yorifumi Satou:** conceptualization (equal), data curation (equal), methodology (equal), project administration (equal), resources (equal), supervision (equal), writing – original draft (equal), writing – review and editing (equal).

## Ethics Statement

Approval of the Research Protocol by an Institutional Reviewer Board: This study was approved by the Ethics Committee of Kumamoto University (approval numbers 1427) and was conducted in accordance with good clinical standards and the Declaration of Helsinki guidelines.

## Consent

All informed consent was obtained from the subject(s) and/or guardian(s).

## Conflicts of Interest

The authors of this manuscript have no conflict of interest. None of the authors of this manuscript is a current editor or editorial board member of Cancer Science. All authors had full access to all of the data in the study and had final responsibility for the decision to submit for publication.

## Supporting information


**Figure S1.** A schematic diagram of PCR primers used for HPV‐16‐positive OPSCC sample genotyping.
**Figure S2.** Representative results of multiplex PCR HPV‐16‐ negative cases.
**Figure S3.** Droplet digital PCR analysis for HPV‐16‐positive candidate samples.

**Table S1.** HPV‐16 sequence of Japanese patients with HPV‐16‐positive cancer.
**Table S2.** Multiplex PCR primer information.
**Table S4.** Clinical information of patients with OSCC.
**Table S5.** Clinical information of patients with OPSCC.

**Table S3.** DNA capture probe information.

## Data Availability

The source data for this study are available from the corresponding author upon reasonable request.
