# Supplementary material for: Robust HPV‐16 Detection Workflow for Formalin‐Fixed Cancer Tissue and Its Application for Oral Squamous Cell Carcinoma
Source: Cancer Med. 2025 Feb 20;14(4):e70544. doi: 10.1002/cam4.70544 (PMC11842277; doi:10.1002/cam4.70544)
Supplement: Supplementary file 1 — Figure S1. A schematic diagram of PCR primers used for HPV‐16‐positive OPSCC sample genotyping. Figure S2. Representative results of multiplex PCR HPV‐16‐ negative cases. Figure S3. Droplet digital PCR analysis for HPV‐16‐positive candidate samples. [file CAM4-14-e70544-s003.pptx]

## Slide 1
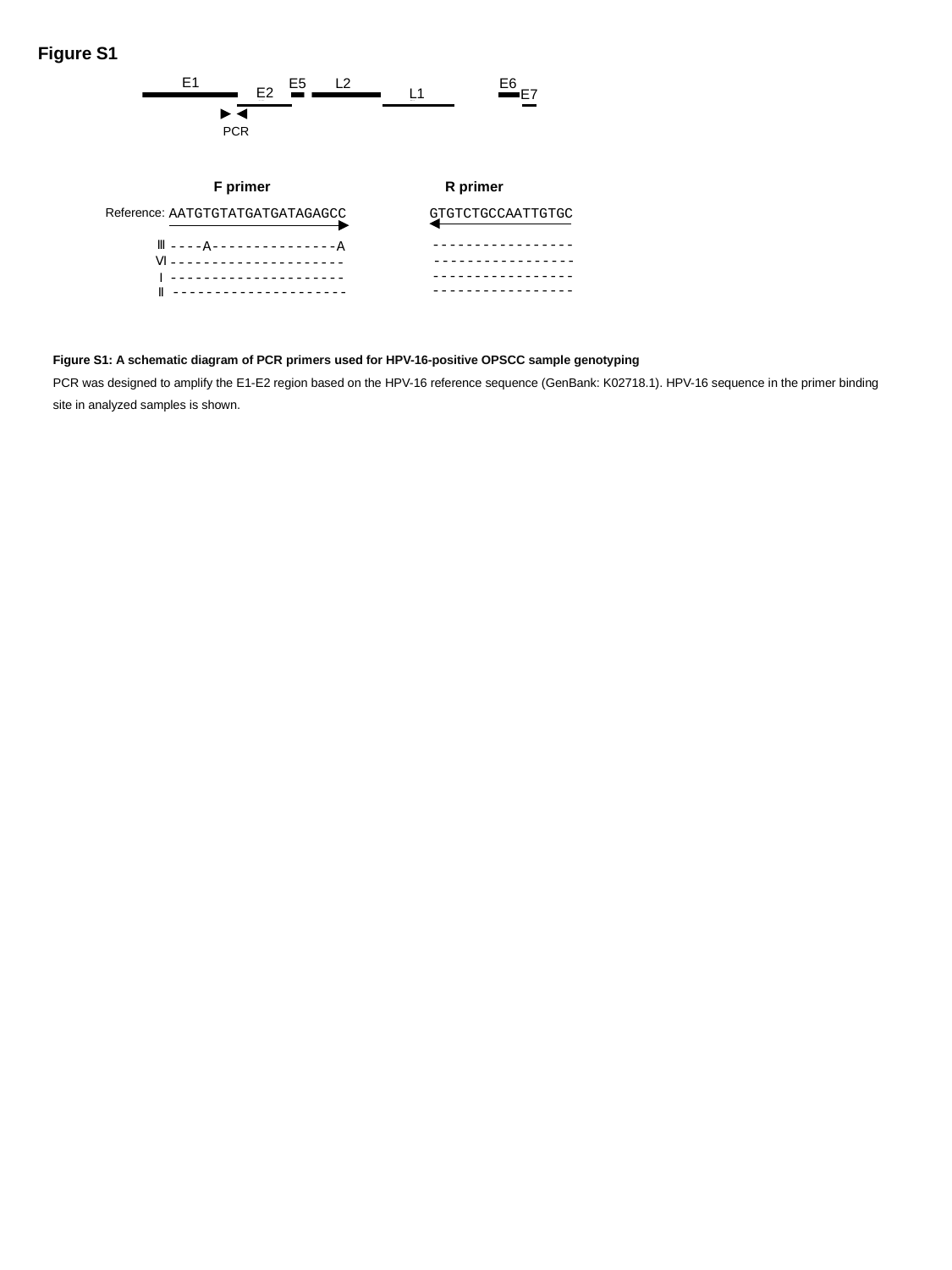

Figure S1
E1
L2
E6
E5
E7
PCR
E2
L1
F primer
R primer
AATGTGTATGATGATAGAGCC
GTGTCTGCCAATTGTGC
Reference:
-
-
-
-
-
-
-
-
-
-
-
-
-
-
-
-
-
Ⅲ
-
-
-
-
A
-
-
-
-
-
-
-
-
-
-
-
-
-
-
-
A
-
-
-
-
-
-
-
-
-
-
-
-
-
-
-
-
-
-
-
-
-
-
-
-
-
-
-
-
-
-
-
-
-
-
-
-
-
-
Ⅵ
-
-
-
-
-
-
-
-
-
-
-
-
-
-
-
-
-
-
-
-
-
-
-
-
-
-
-
-
-
-
-
-
-
-
-
-
-
-
Ⅰ
-
-
-
-
-
-
-
-
-
-
-
-
-
-
-
-
-
-
-
-
-
-
-
-
-
-
-
-
-
-
-
-
-
-
-
-
-
-
Ⅱ
Figure S1: A schematic diagram of PCR primers used for HPV-16-positive OPSCC sample genotyping
PCR was designed to amplify the E1-E2 region based on the HPV-16 reference sequence (GenBank: K02718.1). HPV-16 sequence in the primer binding site in analyzed samples is shown.

## Slide 2
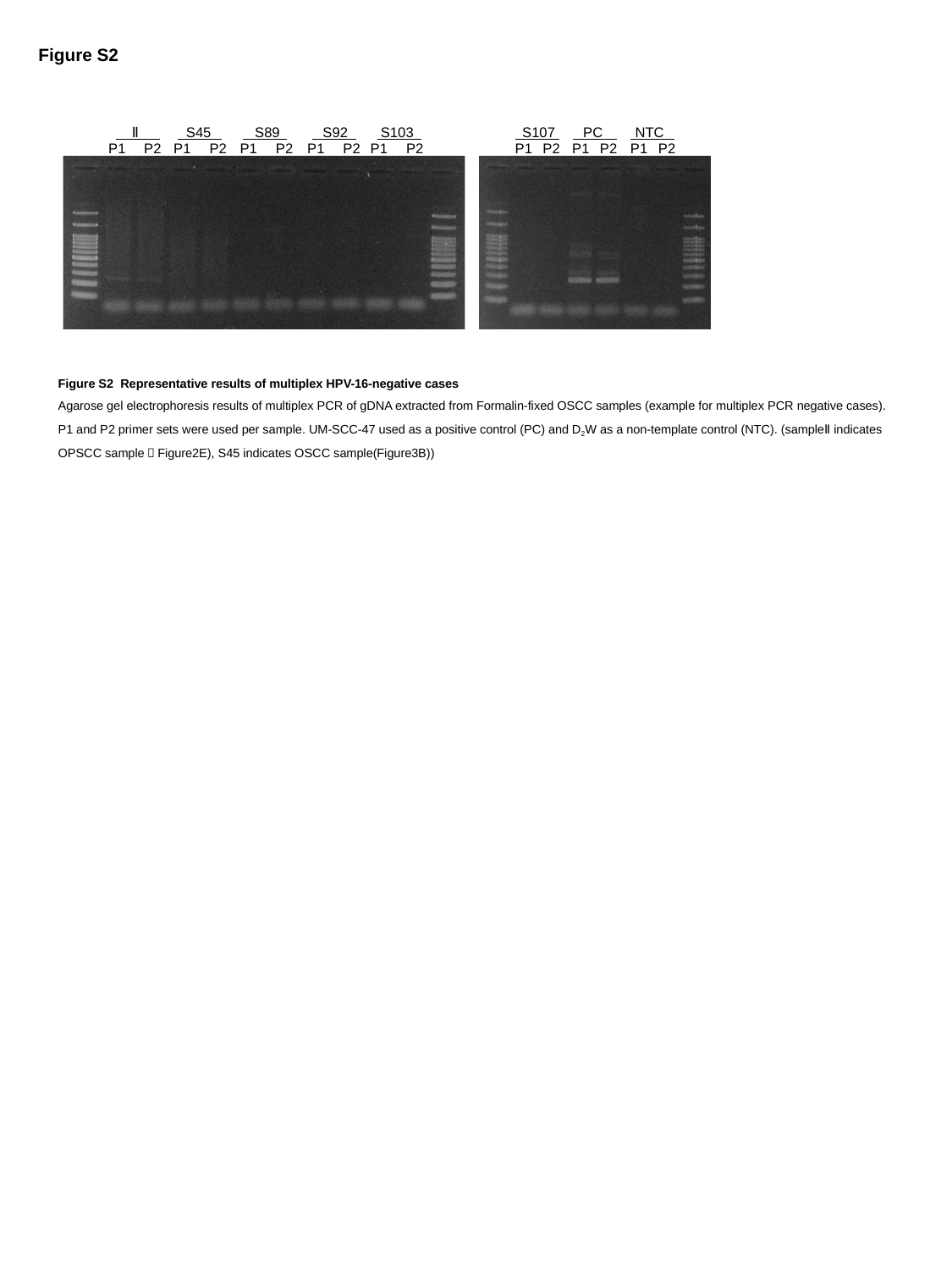

Figure S2
Ⅱ
S45
S89
S92
S103
S107
PC
NTC
P1
P2
P1
P2
P1
P2
P1
P2
P1
P2
P1
P2
P1
P2
P1
P2
Figure S2 Representative results of multiplex HPV-16-negative cases
Agarose gel electrophoresis results of multiplex PCR of gDNA extracted from Formalin-fixed OSCC samples (example for multiplex PCR negative cases). P1 and P2 primer sets were used per sample. UM-SCC-47 used as a positive control (PC) and D2W as a non-template control (NTC). (sampleⅡ indicates OPSCC sample（Figure2E), S45 indicates OSCC sample(Figure3B))

## Slide 3
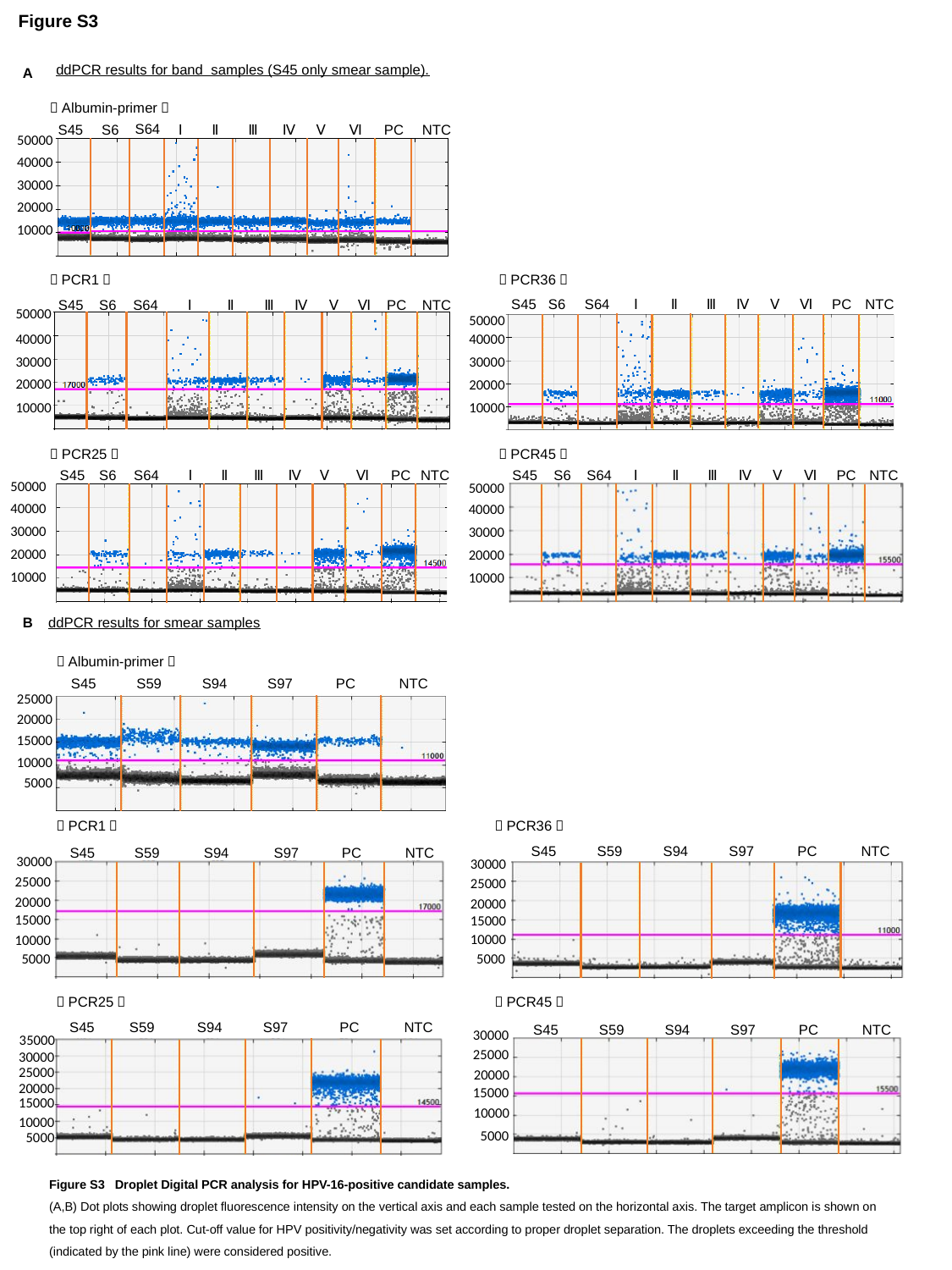

Figure S3
ddPCR results for band samples (S45 only smear sample).
A
［Albumin-primer］
S64
S45
S6
Ⅰ
Ⅱ
Ⅲ
Ⅳ
Ⅴ
Ⅵ
PC
NTC
50000
40000
30000
20000
10000
［PCR1］
［PCR1］
［PCR36］
S45
S6
S64
Ⅰ
Ⅱ
Ⅲ
Ⅳ
Ⅴ
Ⅵ
PC
NTC
S45
S6
S64
Ⅰ
Ⅱ
Ⅲ
Ⅳ
Ⅴ
Ⅵ
PC
NTC
50000
40000
30000
20000
10000
50000
40000
30000
20000
10000
［PCR25］
［PCR45］
S45
S6
S64
Ⅰ
Ⅱ
Ⅲ
Ⅳ
Ⅴ
Ⅵ
PC
NTC
S45
S6
S64
Ⅰ
Ⅱ
Ⅲ
Ⅳ
Ⅴ
Ⅵ
PC
NTC
50000
50000
40000
40000
30000
30000
20000
20000
10000
10000
ddPCR results for smear samples
B
［Albumin-primer］
S59
S45
S94
S97
PC
NTC
25000
20000
15000
10000
5000
［PCR1］
［PCR36］
S59
S45
S94
S97
PC
NTC
30000
25000
20000
15000
10000
5000
S59
S45
S94
S97
PC
NTC
30000
25000
20000
15000
10000
5000
［PCR25］
［PCR45］
S59
S45
S94
S97
PC
NTC
S59
S45
S94
S97
PC
NTC
30000
25000
20000
15000
10000
5000
35000
30000
25000
20000
15000
10000
5000
Figure S3 Droplet Digital PCR analysis for HPV-16-positive candidate samples.
(A,B) Dot plots showing droplet fluorescence intensity on the vertical axis and each sample tested on the horizontal axis. The target amplicon is shown on the top right of each plot. Cut-off value for HPV positivity/negativity was set according to proper droplet separation. The droplets exceeding the threshold (indicated by the pink line) were considered positive.
