# Supplementary material for: Robust HPV‐16 Detection Workflow for Formalin‐Fixed Cancer Tissue and Its Application for Oral Squamous Cell Carcinoma
Source: Cancer Med. 2025 Feb 20;14(4):e70544. doi: 10.1002/cam4.70544 (PMC11842277; doi:10.1002/cam4.70544)
Supplement: Supplementary file 2 — Table S1. HPV‐16 sequence of Japanese patients with HPV‐16‐positive cancer. Table S2. Multiplex PCR primer information. Table S4. Clinical information of patients with OSCC. Table S5. Clinical information of patients with OPSCC. [file CAM4-14-e70544-s002.docx]

Supplementary Information for

**Robust HPV-16 detection workflow for formalin-fixed cancer tissue and its application for oral squamous cell carcinoma**

Shizuka Morodomi, Akiyuki Hirosue, Akhinur Rahman, Kyotaro Nohata, Misaki Matsuo, Omnia Reda, Samiul Alam Rajib, Haruki Saito, Hiroki Takeda, Ryoji Yoshida, Masafumi Nakamoto, Masatoshi Hirayama, Kenta Kawahara , Mitsuyoshi Takatori, Yorihisa Orita, Hideki Nakayama, Yorifumi Satou

Corresponding authors:

Akiyuki Hirosue, DDS, PhD.

1-1-1 Honjo, Chuo-ku, Kumamoto, 860-8556, Japan, Tel: +81-96-373-5288,

**Email:** ahiro711@kumamoto-u.ac.jp

Nakayama Hideki, DDS, PhD.

1-1-1 Honjo, Chuo-ku, Kumamoto, 860-8556, Japan, Tel: +81-96-373-5288,

**Email:** hinakaya@kumamoto-u.ac.jp

This file includes:

I. Supplementary methods

II. Supplementary Tables S1-S5

I. Supplementary methods

**Qualitative analysis (Genotyping PCR)**

Conventional genotyping PCR was performed using HPV-16 positive oropharyngeal cancer. Primers were designed to amplify the E1-E2 region based on the HPV-16 reference sequence (GenBank: K02718.1). PCR was performed using Takara ExTaq (Takara-bio, Japan) 0.125 μL, 10×EX Taq buffer 2.5 μL, 10 mM dNTP 2 μL, 10 uM primer F/R 2.5 μL, from 15 to 90 ng DNA, for a total of 25 μL. PCR conditions were 95 °C 2 min, 98 °C 10 s, 64°C 30 s, 72°C 30 s for 35 cycles, 72°C 5 min, 4°C hold.

**DNA quality check**

The quality of the DNA for all samples was assessed, as reported previously ^14^. The extracted DNA was used to obtain PCR products of different lengths, RAG1 exon2 (200 bp), PLZF exon1 (300 bp), AF4 exon11 (400 bp), and AF4 exon3 (600 bp). Primer sequence information is as follows: RAG1 exon forward: 5’-TGTTGACTCGATCCACCCCA-3’, reverse: 5’- TGAGCTGCAAGTTTGGCTGAA3’, PLZF exon forward: 5’TGCGATGTGGTCATCATGGTG-3’, reverse: 5’ -CGTGTCATTGTCGTCTGAGGC-3’, AF4 exon11 forward: 5’-CCGCAGCAAGCAACGAACC, reverse: 5’ -GCTTTCCTCTGGGCGCTCC -3’, AF4 exon3 forward: 5’- GGAGCAGCATTCATCCAGC-3’and reverse: 5’- CATCCATGGGGCCGGGACATAA-3’. PCR reaction conditions were 95°C for 2 min followed by 35 cycles of 98 °C for 10 s, 60 °C for 30 s, 72 °C for 30 s at 72 °C for 5 min.

**DNA sequencing by Next Generation Sequencing (NGS）**

Multiplex PCR amplicons (pool1& pool2) were used for the NGS library preparation. The PCR product amplicons from multiplex PCR were purified using QIAquick PCR purification Kit (QlAGEN, Germany). The concentration of PCR amplicons was measured by Qubit before DNA Library preparation. The NEB Next Ultra II DNA Library Prep for Illumina Kit (Biolabs, England) was used and the NEBNext Multiplex Oligo for Illumina Kit (Biolabs, England) for library preparation. Briefly, end prep, adapter ligation, library amplification, and indexing steps were performed according to the manufacturer’s recommendations. At the end of the process, the library quality and quantity were checked using a Tape Station 4150 system (Agilent Technologies). Finally, the prepared libraries were equimolarly pooled together, then denatured and sequenced using 300 cycles by MiSeq sequencing systems (Illumina) using nano v2 kit.

**Viral DNA-capture-seq**

Viral DNA-capture-seq with biotinylated HPV-16 probes (Table S3) was performed for cell line, UM-SCC-47, and FFPE sample S6 in a similar way as previously reported ^17, 18^. Briefly, ~1 ug of Genomic DNA extracted from cell line or FFPE tissue was sonicated for 5 cycles (30” on, 90” off) using the Bioruptor Pico Sonication System (Diagenode s.a., Liège, Belgium). The sonicated DNA was used for DNA library preparation according to the manufacturer’s instructions for the NEBNext Ultra II DNA library prep kit and NEBNext Multiplex Oligos for Illumina. The DNA library was quantified using the Tape Station 4150 system (Agilent Technologies). One hundred-twenty-five ng of DNA library from each sample along with 5 ug of human Cot-1 DNA (Invitrogen) was dried up by centrifugal evaporation and then reconstituted in hybridization buffer and hybridization enhancer. The DNA libraries were denatured by incubation for 10 minutes at 95 °C. Multiple biotinylated probes spanning the whole HPV-16 genome were allowed to hybridize for 4 hours at 65 °C with the DNA library. Then, the hybridized DNA libraries were captured using magnetic streptavidin beads (Dynabeads M-280 Streptavidin, Thermo Fisher Scientific). Subsequent wash steps removed unhybridized DNA fragments. After that, the hybridized DNA libraries were amplified by PCR using P5-P7 primers. Finally, the enriched DNA libraries were sequenced using Miseq. The Fasq files generated from Miseq were trimmed by cutadapt, aligned to hg19 & HPV-16 using the BWA-MEM algorithm ^19^, duplicate reads were removed using Picard (<https://github.com/broadinstitute/picard>), and indexed bam files were prepared using samtools for exploration of the coverage of the HPV-16 genome and genomic areas near the HPV-16 integration site in hg19 by using the Integrative Genomics Viewer (IGV) (broad institute).

II. Supplementary Tables S1-S5

Table S1. HPV-16 sequence of Japanese patients with HPV-16-positive cancer

| 1 | LC456191 | 26 | LC456620 | 51 | LC511112 | 76 | LC644188 |
| --- | --- | --- | --- | --- | --- | --- | --- |
| 2 | LC456192 | 27 | LC456621 | 52 | LC644164 | 77 | LC644189 |
| 3 | LC456193 | 28 | LC456622 | 53 | LC644165 | 78 | LC644190 |
| 4 | LC456194 | 29 | LC456623 | 54 | LC644166 | 79 | LC644191 |
| 5 | LC456195 | 30 | LC456624 | 55 | LC644167 | 80 | LC647437 |
| 6 | LC456196 | 31 | LC456625 | 56 | LC644168 | 81 | LC647438 |
| 7 | LC456197 | 32 | LC456626 | 57 | LC644169 | 82 | LC647439 |
| 8 | LC456198 | 33 | LC456627 | 58 | LC644170 | 83 | LC647440 |
| 9 | LC456199 | 34 | LC456628 | 59 | LC644171 | 84 | LC647441 |
| 10 | LC456200 | 35 | LC456629 | 60 | LC644172 | 85 | LC647442 |
| 11 | LC456201 | 36 | LC456630 | 61 | LC644173 | 86 | LC647443 |
| 12 | LC456606 | 37 | LC456631 | 62 | LC644174 | 87 | LC647444 |
| 13 | LC456607 | 38 | LC456632 | 63 | LC644175 | 88 | LC647445 |
| 14 | LC456608 | 39 | LC456633 | 64 | LC644176 | 89 | LC647446 |
| 15 | LC456609 | 40 | LC456634 | 65 | LC644177 | 90 | LC647447 |
| 16 | LC456610 | 41 | LC456635 | 66 | LC644178 | 91 | LC647448 |
| 17 | LC456611 | 42 | LC456636 | 67 | LC644179 | 92 | LC647449 |
| 18 | LC456612 | 43 | LC456637 | 68 | LC644180 | 93 | LC647450 |
| 19 | LC456613 | 44 | LC511105 | 69 | LC644181 | 94 | LC647451 |
| 20 | LC456614 | 45 | LC511106 | 70 | LC644182 | 95 | LC647452 |
| 21 | LC456615 | 46 | LC511107 | 71 | LC644183 | 96 | LC647453 |
| 22 | LC456616 | 47 | LC511108 | 72 | LC644184 | 97 | LC647454 |
| 23 | LC456617 | 48 | LC511109 | 73 | LC644185 | 98 | LC647455 |
| 24 | LC456618 | 49 | LC511110 | 74 | LC644186 | 99 | LC647456 |
| 25 | LC456619 | 50 | LC511111 | 75 | LC644187 | 100 | LC647457 |

101: NC001526

Table S2. Multiplex PCR primer information

| **Primer** | **Forward** | **Reverse** |
| --- | --- | --- |
| 1 | AACCTTTTGTTGCAAGTGTGACT | TCGTCATCTGATATAGCATCCCCT |
| 2 | GGGTACGGGATGTAATGGATGG | AGTGGACTACCCAAATACTTTCGT |
| 3 | CATGCGTTGTTTACTGCACAGG | ATGGCGCCCTTCTACCTGTAA |
| 4 | GAAAGCGAAGACAGCGGGTATG | TTGCTAACATTGCTGCCTTTGC |
| 5 | ACACACTATATGCCAAACACCACT | GAACACGCTAAACTTTGAATGTGTAA |
| 6 | GCTGCATTTGGACTTACACCCA | TGTTTTATACCAATATAATGCTGCTGCT |
| 7 | TCCAATGTGTATGATGATAGAGCCT | AGTGTCTGCCAATTGTGCATATTT |
| 8 | GATGGTACAATGGGCCTACGAT | TTGCTAACATTGCTGCCTTTGC |
| 9 | GGCAAAAATTGTAAAGGATTGTGCA | TTTACCTGTGTTAGCTGCACCA |
| 10 | GGTGTAGAGTTTATGTCATTTTTAACTGC | TCATCTATGTAGTTCCAACAGGGC |
| 11 | GGTTACAACCATTAGCAGATGCC | TGGAAACTCATTAGGAAATGTAAACACC |
| 12 | TGGTACAACTAAAATGCCCTCCA | ACGTTGGCAAAGAGTCTCCATC |
| 13 | CCTTTTTCTCAAGGACGTGGTC | ATTCTTTGATACAGCCAGTGTTGG |
| 14 | TTTATTACAAGGCCAGAGAAATGGG | TCTCCATCAAACTGCACTTCCA |
| 15 | AACCCACAATATAGTAATGAAAAGTGGA | GCACAAAATATGTTTGTATTCCTTCATGAA |
| 16 | GCATCAGTAACTGTGGTAGAGGG | ACGGTTTTGGTATGGGTCGC |
| 17 | GCAGCGACGAAGTATCCTCT | GGGTGTAGTGTTACTATTACAGTTAATCC |
| 18 | GTGGACAGTGCTCCAATCCT | CACGTTGCCATTCACTATCATATGT |
| 19 | ACATGGCATTGGACAGGACATA | AGACAAAAGCAGCGGACGTATT |
| 20 | GTCTATATGACAAACCTTGATACTGCA | TGTAATTAAAAAGCGTGCATGAGTATG |
| 21 | CTGCGTTTAGGTGTTTTATTGGATATATT | GTTTTGCAGAACGTTTGTGTCG |
| 22 | ACAATGCGACACAAACGTTCTG | TAGCTGTGGGAGGCCTTGTT |
| 23 | GGGTTAGGAATTGGAACAGGGT | CAGGTGTGGTATCAGTTGAAGTAGT |
| 24 | AGTTTCTTTAGTGGACGAAACTAGTTT | TGTGTACTAATAGTGGATGATGAAAGTG |
| 25 | CCCACTTTCACTGACCCATCTG | GAGTGGTTACAAAAGCAGGGTC |
| 26 | CAGTGGCACGCCTAGGATTATA | CTGTACCTAATGCCAGTACGCC |
| 27 | AGCTCCAGATCCTGACTTTTTGG | CATTATTAATAGAAGTAGGTGAGGCTGC |
| 28 | CTGCAGAAGAAATAGAATTGCAAACTAT | CAGTTGTATTAATGGGTATATCAGGACC |
| 29 | CCTGCAAATACAACAATTCCTTTTGG | AGTAGACAGTGGCCTCGCTA |
| 30 | GGGTCTCCACAATATACAATTATTGCT | GGAAAATAGGGATGTCCAACTGC |
| 31 | GGATGAATATGTTGCACGCACA | ACGACCTACCTCAACACCTACA |
| 32 | TTGGTTTTCCTGACACCTCATTTT | CCAGTGTTCCCCTATAGGTGGT |
| 33 | ACACAGAAAATGCTAGTGCTTATGC | GCCTGTAATGTAGTAAAGTCCATAGC |
| 34 | TTGTCCACCATTAGAGTTAATAAACACA | CCAACAGCACCAGCCCTATTAA |
| 35 | TGTCAGAACCATATGGCGACAG | CCATTATTGTGGCCCTGTGCTC |
| 36 | TCAAATTATTTTCCTACACCTAGTGGTT | ACTGTAAATCATATTCCTCCCCATGT |
| 37 | GCTGCCATATCTACTTCAGAAACTAC | TTTTGACAAGCAATTGCCTGGG |
| 38 | GGACTGGAATTTTGGTCTACAACC | CTTTTCGTTTCCCTAATGTAAATTTTGGT |
| 39 | TGCAGACCTAGATCAGTTTCCTTT | ACCATACATACAAACACACATACAACTTA |
| 40 | ACCTCATCTACCTCTACAACTGCT | TGAATAACCACAACACAATTAGTAGGT |
| 41 | TCAATGCTTGTGTAACTATTGTGTCA | GGAAACGTACAAGTTTAAACTATAGTTGC |
| 42 | TTCGGTTGCATGCTTTTTGGC | CAAAAATATGTGCCTAACAGCGGT |
| 43 | TGACTCACTATGTACATTGTGTCATATAAA | AAGTTGCTTGTAAATGTGTAACCCA |
| 44 | ACTAAGGGCGTAACCGAAATCG | TGGACTCCCATCTCTATATACTATGCA |
| 45 | AAGCAACAGTTACTGCGACGT | TTTTCTTCAGGACACAGTGGCT |
| 46 | AGAACAGCAATACAACAAACCGT | AGATCAGTTGTCTCTGGTTGCA |
| 47 | CCCAGCTGTAATCATGCATGGA | TCCAAAGTACGAATGTCTACGTGT |

Table S3. DNA capture probe information (Excel sheet attached)

Table S4: Oral cancer patients’ clinical information

|  | **Age** | **Gender** | **Sampling part** | **cTNM classification** | **Smoking** | **Alcohol** | **p16 staining** |
| --- | --- | --- | --- | --- | --- | --- | --- |
| 1 | 70 | M | Tongue | T2N0M0 | ー | ー | A |
| 2 | 68 | M | Floor of the mouth | T1N0M0 | ー | ＋ | A |
| 3 | 78 | F | Maxillary gingiva | T4aN1M0 | ー | ー | A |
| 4 | 88 | M | Hard plate | T4bN0M0 | ー | ー | A |
| 5 | 43 | F | Tongue | TisN0M0 | ー | ＋ | A |
| 6 | 74 | M | Tongue | T2N0M0 | ー | ー | A |
| 7 | 69 | M | Tongue | T2N0MO | ー | ＋ | A |
| 8 | 84 | F | Maxillary gingiva | T2N0M0 | ー | ＋ | A |
| 9 | 84 | M | Floor of the mouth | T2N2bM0 | ー | ＋ | A |
| 10 | 38 | F | Tongue | T3N2bM0 | ＋ | ＋ | A |
| 11 | 60 | M | Maxillary gingiva | T3N0M0 | ＋ | ー | A |
| 12 | 64 | M | Buccal mucosa | T2N0M0 | ー | ＋ | A |
| 13 | 56 | M | Tongue | T2N0M0 | ＋ | ＋ | A |
| 14 | 84 | F | Maxillary gingiva | T2N0M0 | ー | ＋ | A |
| 15 | 73 | F | Tongue-Floor of the mouth | T1N2aM0 | ー | ー | NA |
| 16 | 44 | M | Tongue | T4aN2bM0 | ＋ | ＋ | NA |
| 17 | 78 | M | Maxillary gingiva | T4bN0M0 | ー | ＋ | NA |
| 18 | 80 | F | Tongue | T1N0M0 | ー | ー | NA |
| 19 | 69 | M | Buccal mucosa | T1N0MO | ー | ー | NA |
| 20 | 64 | M | Mandibular gingiva | T4bN2bM0 | ー | ー | NA |
| 21 | 81 | F | Hard palate | T4aN0M0 | ー | ー | NA |
| 22 | 79 | M | Maxillary gingiva | T4aN0M0 | ー | ＋ | NA |
| 23 | 48 | F | Tongue | T2N1M0 | ー | ー | NA |
| 24 | 95 | F | Mandibular gingiva | ー | ー | ー | NA |
| 25 | 67 | M | Tongue | T4aN2bM0 | ー | ＋ | NA |
| 26 | 69 | M | Tongue | T2N0M0 | ー | ＋ | NA |
| 27 | 53 | M | Tongue | T4aN2cM1 | ＋ | ＋ | NA |
| 28 | 74 | M | Tongue | T4aN2bM0 | ー | ー | NA |
| 29 | 82 | F | Mandibular gingiva | rT1N0M0 | ー | ー | NA |
| 30 | 84 | M | Tongue | T3N2bM0 | ー | ＋ | NA |
| 31 | 76 | F | Buccal mucosa | T3N2bM0 | ー | ー | NA |
| 32 | 76 | F | Hard palate | T3N2bM0 | ー | ー | NA |
| 33 | 85 | F | Buccal mucosa | T3N2bM0 | ー | ー | NA |
| 34 | 72 | F | Lower gingiva | T4aN2cM0 | ー | ー | NA |
| 35 | 72 | F | Buccal mucosa | T4aN2cM0 | ー | ー | NA |
| 36 | 93 | M | Floor of the mouth | T2N0M0 | ー | ＋ | NA |
| 37 | 67 | M | Tongue | T4aN2cM0 | ＋ | ー | NA |
| 38 | 89 | F | Mandibular gingiva | T2N2bM0 | ー | ー | NA |
| 39 | 84 | F | Mandibular gingiva | T4aN0M0 | ー | ー | NA |
| 40 | 68 | M | Tongue | TisN0M0 | ー | ＋ | NA |
| 41 | 77 | M | Maxillary gingiva | T4aN0M0 | ー | ー | NA |
| 42 | 62 | F | Lower gingiva | T4aN2cM0 | ー | ー | NA |
| 43 | 66 | M | Buccal mucosa | T1N0M0 | ー | ー | NA |
| 44 | 89 | M | Buccal mucosa | ー | ー | ー | NA |
| 45 | 52 | F | Tongue | T1N0M0 | ＋ | ＋ | ND |
| 46 | 81 | M | Tongue | T2N2bM0 | ー | ＋ | ND |
| 47 | 66 | F | Maxillary Gingiva | T4aN0M0 | ー | ー | ND |
| 48 | 81 | F | Buccal mucosa | T4aN2bM0 | ー | ー | ND |
| 49 | 89 | F | Tongue | ー | ー | ー | ND |
| 50 | 74 | M | Tongue | ー | ー | ＋ | ND |
| 51 | 53 | M | Tongue | T4aN2bM0 | ＋ | ー | ND |
| 52 | 62 | F | Gingiva | T3N0M0 | ー | ー | ND |
| 53 | 65 | M | Mandibular gingiva | T4aN0M0 | ー | ＋ | ND |
| 54 | 85 | F | Tongue | T2N0M0 | ー | ー | ND |
| 55 | 73 | F | Tongue | T4aN0M0 | ー | ー | ND |
| 56 | 91 | F | Tongue | T1N0M0 | ー | ー | ND |
| 57 | 72 | M | Tongue | T1N0M0 | ー | ＋ | ND |
| 58 | 84 | F | Tongue | rT3N0M0 | ー | ー | ND |
| 59 | 80 | M | Buccal mucosa | T3N3bM0 | ＋ | ー | ND |
| 60 | 89 | M | Buccal mucosa | T4aN1M0 | ー | ー | ND |
| 61 | 61 | M | Mandibular gingiva | T2N0M0 | ー | ー | ND |
| 62 | 73 | F | Mandibular. gingiva | T2N0M0 | ー | ー | ND |
| 63 | 87 | F | Tongue | rT1N0M0 | ー | ー | ND |
| 64 | 54 | F | Tongue | T1N0M0 | ー | ＋ | ND |
| 65 | 69 | F | Maxillary Gingiva | T3N0M0 | ー | ー | ND |
| 66 | 82 | F | Maxillary Gingiva | T2N1M0 | ー | ＋ | ND |
| 67 | 87 | F | Tongue | T1N0M0 | ー | ー | ND |
| 68 | 64 | F | Tongue | T2N0M0 | ー | ー | ND |
| 69 | 82 | F | Maxillary Gingiva | T1N0M0 | ー | ー | ND |
| 70 | 45 | F | Tongue | T3N2b-2cM0 | ー | ＋ | ND |
| 71 | 81 | M | Tongue | rT1N0M0 | ー | ＋ | ND |
| 72 | 72 | M | Buccal mucosa | T1N0M0 | ー | ＋ | ND |
| 73 | 79 | F | Floor of mouth | TisN0M0 | ー | ー | ND |
| 74 | 64 | F | Tongue | T2N0M0 | ー | ー | ND |
| 75 | 88 | F | Mandibular gingiva | ー | ー | ー | ND |
| 76 | 86 | F | Buccal mucosa | rT4aN0M0 | ー | ー | ND |
| 77 | 74 | M | Tongue | T3N0M0 | ー | ＋ | ND |
| 78 | 71 | M | Tongue | T2N1M0 | ー・ | ＋ | ND |
| 79 | 49 | F | Floor of mouth | TisN0M0 | ー | ＋ | ND |
| 80 | 73 | M | Tongue | T4aN2bM0 | ＋ | ー | ND |
| 81 | 85 | F | Lip-Buccal mucosa | T3N0M0 | ー | ー | ND |
| 82 | 87 | F | Tongue | T1N0M0 | ー | ー | ND |
| 83 | 86 | M | Tongue | T2N0M0 | ー | ＋ | ND |
| 84 | 86 | F | Floor of mouth | T1N0M0 | ー | ー | ND |
| 85 | 58 | F | Tongue | ー | ＋ | ＋ | ND |
| 86 | 73 | M | Maxillary Gingiva | T4bN1M0 | ー | ＋ | ND |
| 87 | 74 | M | Mandibular Gingiva | T4aN0M0 | ー | ー | ND |
| 88 | 84 | F | Mandibular Gingiva | T2N0M0 | ー | ー | ND |
| 89 | 85 | F | Mandibular Gingiva | T2N0M0 | ー | ー | ND |
| 90 | 64 | F | Buccal mucosa | T4aN2bM0 | ＋ | ー | ND |
| 91 | 84 | F | Mandibular gingiva | T4aN0M0 | ー | ー | ND |
| 92 | 55 | F | Tongue | ー | ＋ | ー | ND |
| 93 | 85 | F | Mandibular gingiva | ー | ー | ー | ND |
| 94 | 84 | M | Tongue | T3N2bM0 | ー | ー | ND |
| 95 | 83 | F | Buccal mucosa | T4aN2bM0 | ー | ー | ND |
| 96 | 77 | M | Buccal mucosa | T2N0M0 | ー | ー | ND |
| 97 | 59 | M | Tongue | T4aN1M0 | ＋ | ＋ | ND |
| 98 | 44 | M | Tongue | TisN0M0 | ＋ | ＋ | ND |
| 99 | 90 | F | Tongue | ー | ー | ー | ND |
| 100 | 93 | F | Maxillary gingiva | T3N0MO | ー | ー | ND |
| 101 | 65 | M | Mandibular gingiva | T4aN0M0 | ＋ | ー | ND |
| 102 | 67 | F | Tongue | TisN0M0 | ー | ー | ND |
| 103 | 68 | F | Tongue | T1N0M0 | ＋ | ー | ND |
| 104 | 48 | M | Tongue | T1N0M0 | ＋ | ＋ | ND |
| 105 | 83 | F | Maxillary Gingiva | T2N0M0 | ー | ー | ND |
| 106 | 95 | F | Mandibular gingiva | ー | ー | ー | ND |
| 107 | 83 | M | Lower gingiva | T4bN2bM0 | ー | ー | ND |
| 108 | 83 | F | Buccal mucosa | T4aN2bM0 | ー | ー | ND |
| 109 | 91 | F | Tongue | T4bN0M0 | ー | ー | ND |
| 110 | 85 | F | Tongue | TisN0M0 | ー | ー | ND |
| 111 | 73 | M | Floor of mouth | T1N0M0 | ＋ | ＋ | ND |
| 112 | 79 | M | Floor of mouth | T1N0M0 | ＋ | ＋ | ND |
| 113 | 61 | F | Tongue | T2N0M0 | ー | ー | ND |
| 114 | 58 | M | Lip | T0N0M1  (Extra regional transfer) | ＋ | ー | ND |
| 115 | 61 | M | Tongue | T2N0M0 | ＋ | ー | ND |
| 116 | 75 | M | Tongue | T2N2bM0 | ＋ | ー | ND |
| 117 | 85 | F | Tongue | T4aN2bM0 | ー | ー | ND |
| 118 | 43 | F | Tongue | TisN0M0 | ＋ | ＋ | ND |
| 119 | 60 | M | Floor of mouth | T2N0M0 | ＋ | ー | ND |
| 120 | 63 | M | Mandibular gingiva | T4aN2bM0 | ＋ | ー | ND |
| 121 | 84 | F | Mandibular gingiva | T4aN2bM0 | ＋ | ー | ND |
| 122 | 81 | F | Tongue | T1N0M0 | ー | ー | ND |
| 123 | 72 | F | Tongue | T2N0M0 | ー | ー | ND |
| 124 | 72 | F | Tongue | T2N0M0 | ＋ | ー | ND |
| 125 | 85 | M | Tongue | T2N0M0 | ＋ | ー | ND |
| 126 | 86 | F | Maxillary gingiva | rT3N0M0 | ＋ | ー | ND |
| 127 | 70 | M | Maxillary gingiva | T4aN2bM0 | ー | ー | ND |

Table S5: Oropharyngeal cancer patients’ clinical information

|  | **Age** | **gender** | **parts** | **cTNM classification** | **Smoking** | **Alcohol** | **p16 staining** |
| --- | --- | --- | --- | --- | --- | --- | --- |
| Ⅰ | 58 | M | lateral wall | T3N0M0 | ー | ＋ | A |
| Ⅱ | 61 | M | posterior wall | T4bN2cM0 | ー | ＋ | A |
| Ⅲ | 54 | M | base of tongue | T3N2cM0 | ー | ー | A |
| Ⅳ | 71 | F | base of tongue | T3N1M0 | ー | ＋ | A |
| Ⅴ | 64 | M | tonsillar fossa | T2N2bM0 | ＋ | ＋ | A |
| Ⅵ | 68 | M | base of tongue | T1N2bM0 | ー | ー | A |
